# Impact of hypothermia alert device (BEMPU) on improvement of duration of Kangaroo Mother Care (KMC) provided at home: parallel-group randomized control trial

**DOI:** 10.1038/s41598-023-29388-0

**Published:** 2023-03-16

**Authors:** Somashekhar Marutirao Nimbalkar, Viral Thakorbhai Patel, Dipen Vasudev Patel, Ajay Gajanan Phatak

**Affiliations:** 1 Department of Neonatology, Pramukhswami Medical College, Bhaikaka University, Karamsad, Gujarat 388325 India; 2 Central Research Services, Bhaikaka University, Karamsad, Gujarat 388325 India

**Keywords:** Physiology, Health care, Medical research

## Abstract

The objective of the study was to determine if using the hypothermia-detecting bracelet (named BEMPU) improves the duration of Kangaroo Mother Care (KMC) at home by one hour. This parallel-group randomized controlled trial was conducted at a step-down nursery of a teaching hospital. Neonates between 1000 and 2000 g were randomized to BEMPU and control groups at the time of discharge. BEMPU was applied at the wrist of each newborn in the BEMPU (intervention) group. Parents were advised to keep the BEMPU in place till 4 weeks post-discharge. The BEMPU generates a beep sound as an alarm when a newborn's temperature drops below 36.5 °C. Parents in both groups were trained to provide KMC at home. Parents in the BEMPU group received the "KMC chart" and "BEMPU beep chart," while the control group received the "KMC chart" only. In the "KMC chart," parents entered information about KMC hours on a real-time basis, and in the "BEMPU beep chart," they entered information about alarm beeps from BEMPU on a real-time basis till 4 weeks post-discharge. Independent samples t-test was used to compare mean KMC hours between the two groups. A total of 128 neonates participated in the study (64 in BEMPU and 64 in Control groups). The mean(SD) gestational age for the BEMPU group was 34.04(2.84) weeks vs 34.75(2.70) weeks for the control group. In BEMPU group, mean(SD) daily time spent doing KMC was significantly higher in 1st week [4.78(2.93) vs. 3.22(2.44) h, *p* = 0.003], in 2nd week [4.52(3.43) vs. 2.84(2.95) h, *p* = 0.008], in 3rd week [4.23(3.71) vs. 2.30(2.70) h, *p* = 0.003], in 4th week [3.72(3.30) vs. 1.95(2.65) h, *p* = 0.003] as compared to control group. BEMPU improved the daily duration of KMC hours at home compared to the control group over four weeks.

*Clinical Trial Registration*: This trial is registered at Clinical Trials Registry India with registration number: CTRI/2018/08/015154 and accessible at http://ctri.nic.in/Clinicaltrials/pdf_generate.php?trialid=27600&EncHid=&modid=&compid=%27,%2727600det%27 Registered on 01/08/2018.

The World Health Organization (WHO) recognizes neonatal hypothermia as one of the major risk factors for morbidity and mortality in newborns^1^. A thermoneutral environment ensures optimum temperature to maintain a minimal basal metabolic rate ensuring that the neonate expends energy for growth and not for maintaining temperature^1^.

In India, the prevalence of hypothermia varies in normal newborns. It has been reported recently in community, and hospital settings at around 31% and 32%, respectively, but these mainly included normal-weight newborns^2,3^. The prevalence of neonatal hypothermia is higher for low birth weight infants (LBWI) and preterm newborns. Twenty-seven million infants are born in India every year, out of which 8 million (30%) are Low Birth Weight Infants [LBWI]^4^. Preterm and LBWI are more prone to hypothermia and their immature temperature regulation may result in consequences such as apnea, hypoglycemia, and poor weight gain^5^. Neonatal hypothermia prevention can reduce up to 42% of neonatal deaths^6^. A hypothermic neonate is susceptible to an increase of 11% in late-onset sepsis for every 1ºC drop in temperature below 36 °C^7^.

There are various measures to keep the LBWI warm, e.g., radiant warmer, incubators, and Kangaroo Mother Care (KMC). KMC, apart from maintaining temperature, has even more important benefits, such as reducing mortality, promoting breastfeeding, improving weight gain, reducing the length of hospital stay, etc.^8^.

The hypothermia alert device (Newborn Hypothermia Monitor) innovated by BEMPU Health Pvt, Ltd, Bengaluru, India, is a neonatal bracelet that is to be worn on the wrist. It is called BEMPU or TempWatch. The BEMPU continuously monitors the skin temperature of the neonates by its temperature sensor, which remains in continuous contact with the skin of the wrist. Its shelf-life is 30 days. Whenever the temperature drops below 36.5 °C, the BEMPU makes a beeping sound (alarm) and shows an orange light alerting the parents to take adequate measures to provide warmth to the infant by giving KMC^9–11^ (Fig. 1).

**Figure 1:**
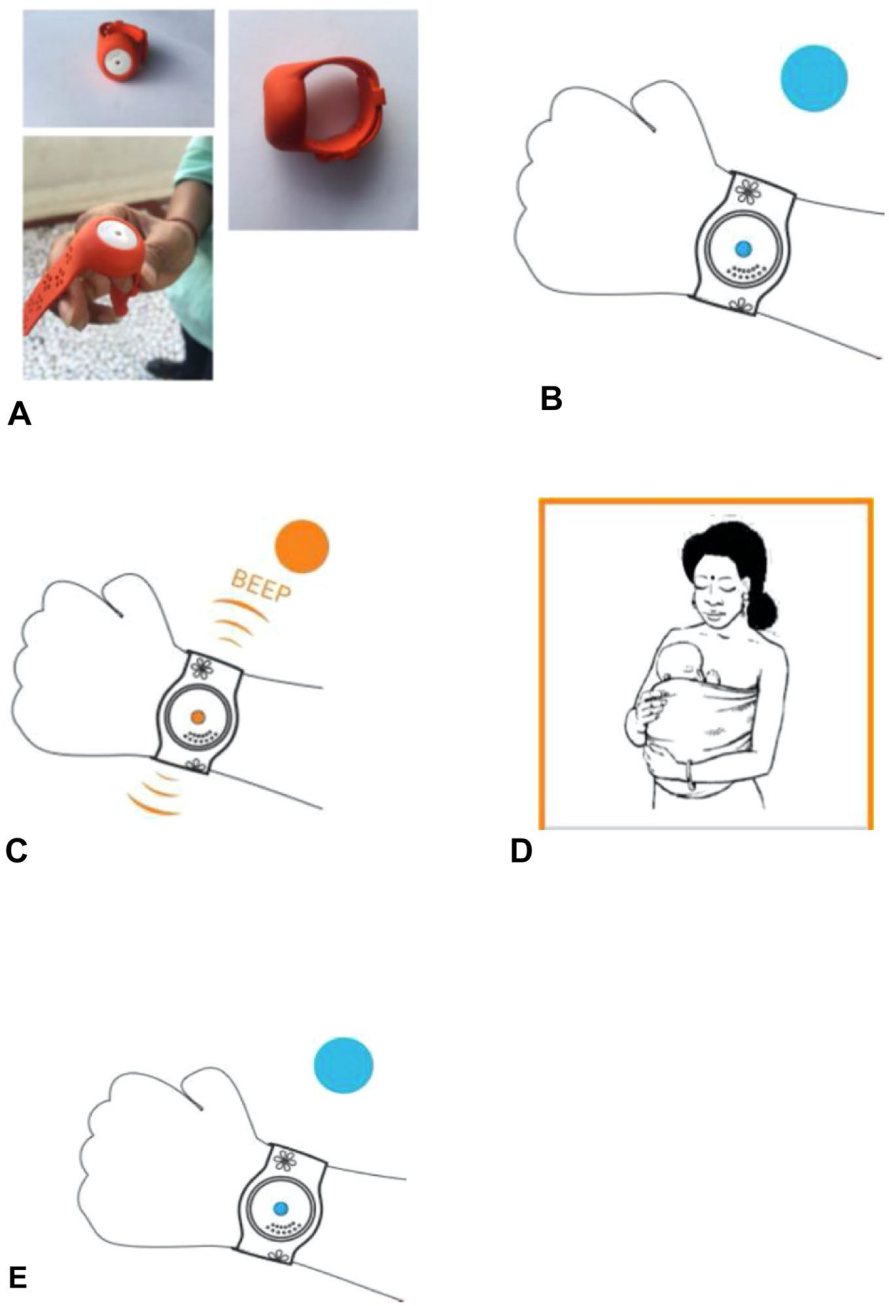
BEMPU bracelet (**A**) BEMPU device, (**B)** BEMPU flashes a blue light when neonate has a normal temperature, (**C**) BEMPU flashes an orange light and beep alarms when the neonate is hypothermic. (**D**) Mother takes action to manage hypothermia by giving KMC, (**E**) Once the temperature reaches normal (≥ 36.5 °C), BEMPU flashes blue light again.

Despite the primacy of KMC as a therapy for the improvement of outcomes for LBWI, there are few innovations that can promote KMC by motivating caregivers to increase the quality of KMC or impressing upon them the need for KMC. Despite standardized guidelines available in India for KMC implementation, its utilization is suboptimal in neonatal intensive care units (NICU) and at the community level^12–14^.

Various strategies, including KMC champions and formative research, are suggested to improve KMC implementation in the hospital and community settings^15,16^. A randomized control trial to study the impact of the BEMPU on KMC hours was conducted to assess whether using the BEMPU around the time of discharge and at home for a total duration of 28 days increased the number of KMC hours by at least one hour.

## Result

**Figure 2:**
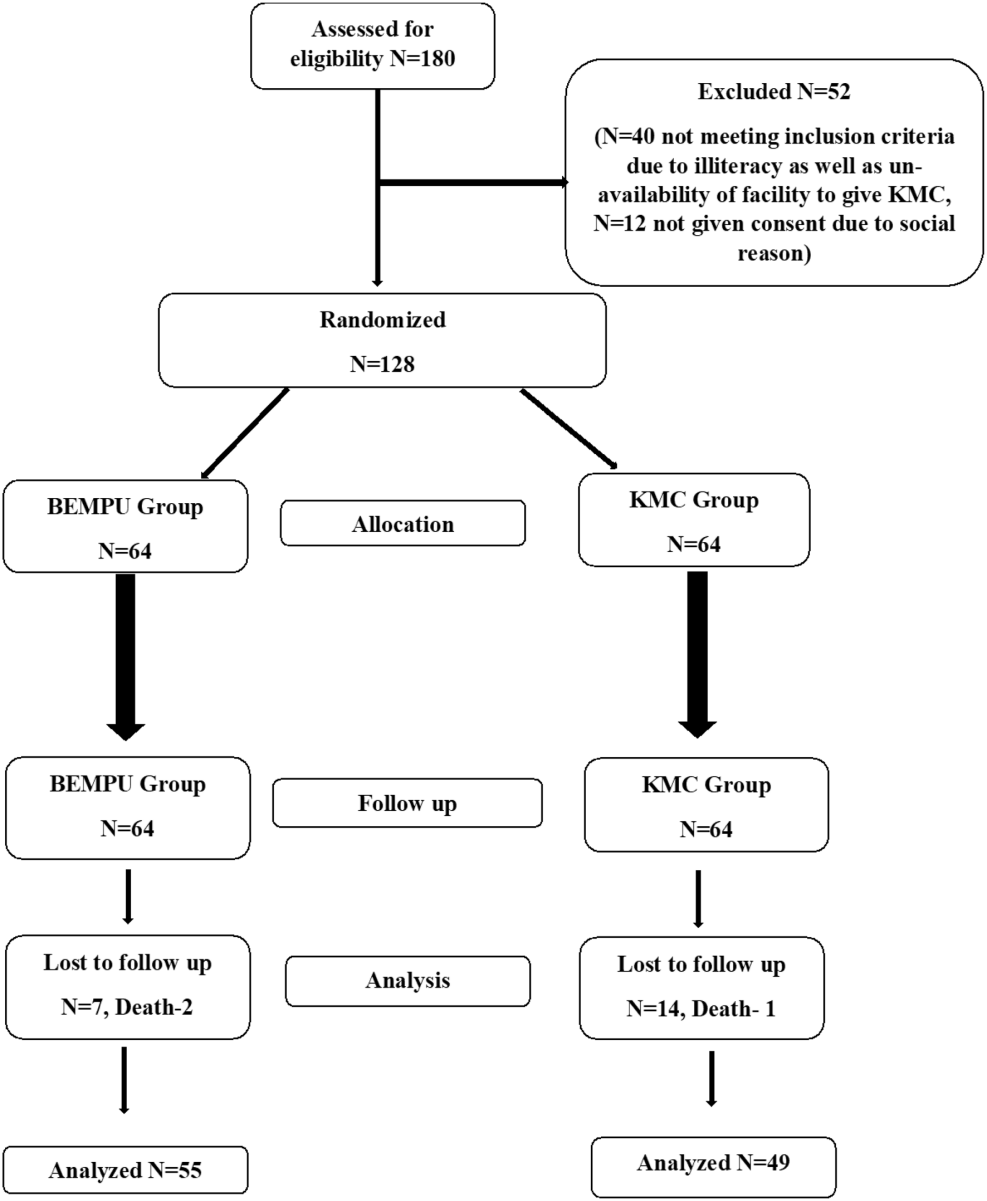
CONSORT flow diagram of study participants.

From August 2018 to June 2019, 128 newborns were enrolled and assigned to one of two groups (CONSORT diagram Fig. 2 ).

There were no differences in the demographic or other clinical parameters between the groups (Table 1). The common diagnoses in both groups were respiratory distress, sepsis, jaundice and perinatal asphyxia.

**Table 1:** Baseline characteristics of study participants.

	BEMPU (N = 64)		Control (N = 64)		*p* Value
	Mean (SD)	Median [IQR]	Mean (SD)	Median [IQR]	
Gestational age (weeks)	34.04 (2.84)		34.75 (2.70)		0.15
Birth weight (g)*		1645 [1315,1855]		1720 [1500,1900]	0.24
Age at enrolment (days)*		12 [6,25.50]		10[4, 20]	0.19
Weight on discharge (g)	1730 (175)		1743 (164)		0.64
Day of life when KMC was started*		9[5, 20]		8.5 [4,18.5]	0.52
Chronological age on discharge*		16[8,29.5]		13 [6,22.5]	0.07
	n(%)		n(%)		
Gender of newborn (Female)	29 (45.31%)		25 (39.06%)		0.47
Appropriate for gestational age	35 (54.69%)		35 (54.69%)		1.0
Vaginal delivery	43 (67.19%)		31 (48.44%)		0.03
Respiratory Distress	27 (42.19%)		25 (39.06%)		0.72
Sepsis	33 (51.56%)		21 (32.81%)		0.03
Perinatal asphyxia	9 (14.06%)		8 (12.5%)		0.79
Jaundice	27 (42.19%)		28 (43.75%))		0.86

*Not normally distributed.

Out of 64 participants from the BEMPU group, 55 neonates completed their four-week follow-up. Two neonates died; one died two weeks after of discharge, and another died three weeks after discharge. Both completed their first visits. Out of 64 from the BEMPU group, seven participants were lost to complete follow-up. While out of 64 participants from the Control group, 49 completed their four weeks follow-ups, one baby died after one week of discharge, and 14 participants were completely lost to follow-up after discharge. We tried to reach them through telephonic communication, but they could not attend the hospital for follow-up.

There was 100% compliance in the filling of the KMC chart as well as the BEMPU chart (55 participants among the BEMPU group and 49 participants among the Control group).

**Figure 3:**
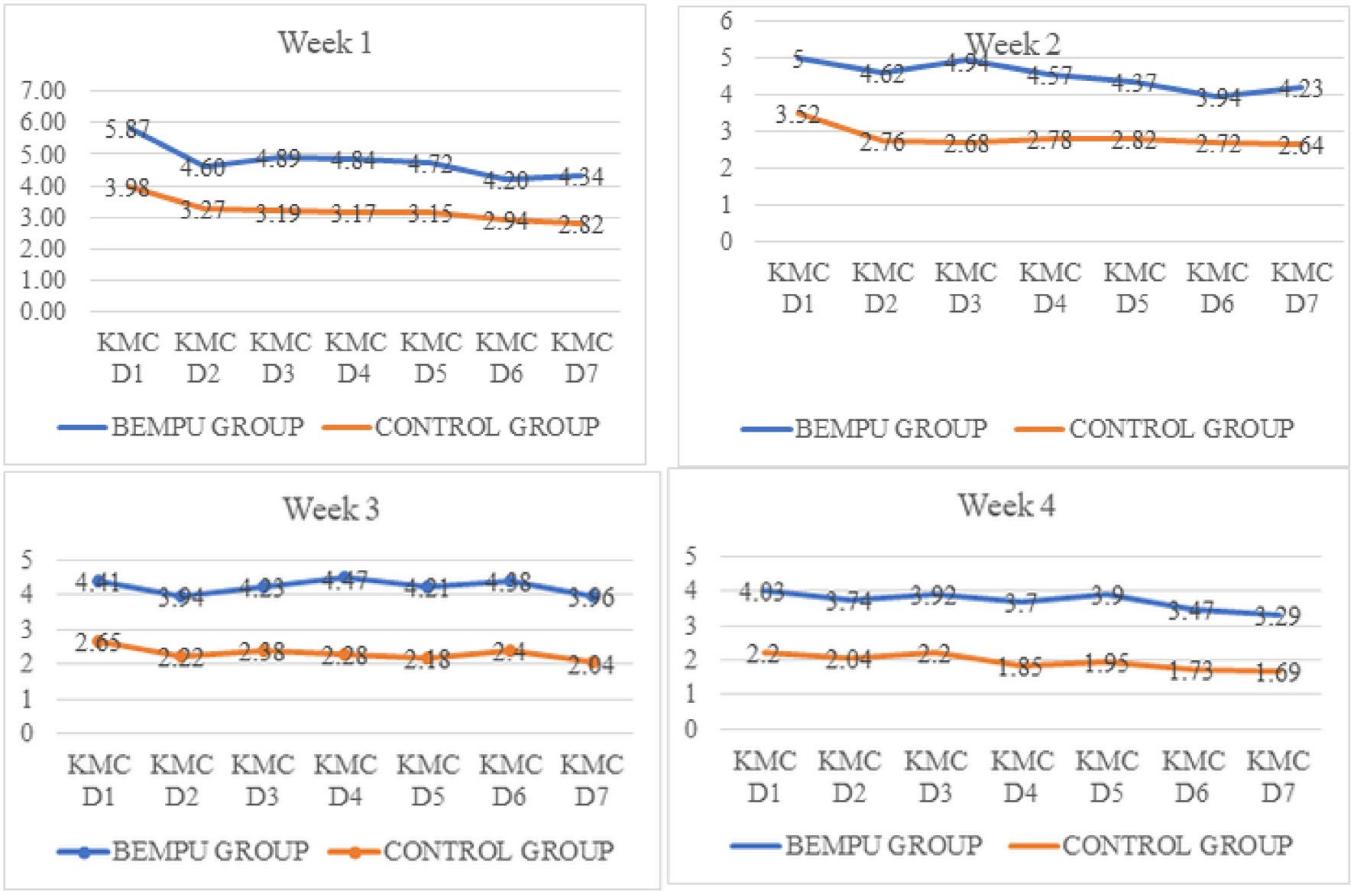
Line chart of daily mean KMC hours over 4 weeks.

**Figure 4:**
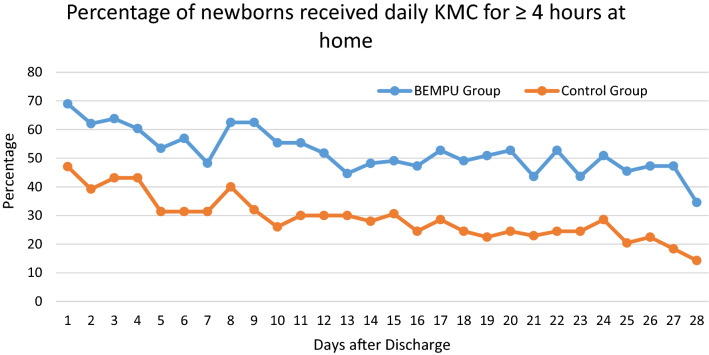
Percentage of newborns received daily KMC for ≥ 4 h at home over 4 weeks.

Daily KMC hours were significantly higher in the BEMPU group versus the control group (Fig. 3). As expected, with increasing maturity and an increase in the child's weight over time, KMC hours were reduced in both groups. However, the significant difference between the BEMPU and Control groups persisted. Parents in the BEMPU group used KMC more frequently as compared to the control group. In the BEMPU group, the mean (SD) daily time spent in hours (h) doing KMC was significantly higher across all four weeks as compared to the control group (Table 2). In addition, the proportion of newborns who received total cumulative of at least four hours of KMC per day in the BEMPU group was significantly more as compared to the control group across all four weeks (all p < 0.05) (Fig. 4).

**Table 2:** Daily KMC hours (weekly).

	BEMPU group Mean (SD) N (%)			Control group Mean (SD) N	P1*	P2*	P3*
	Overall	No Beep	Beeps				
WEEK1	4.78(2.93) 58	3.21(1.69) 14(24.14)	5.28(3.07) 44(75.86)	3.22(2.44) 51	0.003	0.01	0.99
WEEK2	4.52(3.43) 56	3.06(1.69) 16(28.57)	5.11(3.77) 40(71.43)	2.84(2.95) 50	0.008	0.04	0.78
WEEK3	4.23(3.71) 55	3.10(3.51) 21(38.18)	4.92(3.72) 34(61.82)	2.30(2.70) 49	0.003	0.07	0.30
WEEK4	3.72(3.30) 55	2.87(2.93) 28(50.91)	4.61(3.49) 27(49.09)	1.95(2.65) 49	0.003	0.050	0.16

* = *p*-value, P1 = BEMPU group (Overall) vs. Control group, P2 = No Beep vs. Beeps.

P3 = No Beep vs. Control group.

The mean(SD) weight gain over four weeks in the BEMPU group was similar as compared to the control group [756(249) g vs 774(265) g, p = 0.73]. A similar trend was observed in mean(SD) length [4.28(2.9) cm vs 4.41(2.52) cm, p = 0.84]. However, the mean(SD) gain in head circumference over four weeks was significantly less in the BEMPU group as compared to the control group [3.42(0.91) mm vs. 3.99(1.32) mm; p = 0.01].

The study showed an additional correlation between events of drop in skin temperature prompted by beeps from BEMPU with KMC hours within the BEMPU group. Mean(SD) KMC hours were higher in the newborns in whom the BEMPU beeped than in newborns in whom the BEMPU did not beep over four weeks. However, the mean(SD) KMC hours were similar in no beep group as compared to the control group across four weeks (Table 2). No adverse event was reported while using BEMPU.

## Discussion

The current RCT was conducted in LBW newborns to study the impact of BEMPU (a hypothermia-detecting bracelet) on KMC hours at home. The newborns in the BEMPU group received KMC for a longer duration as compared to the control group for four weeks after discharge.

Despite thermal care being considered as an essential component of the care of newborns, the incidence of neonatal hypothermia is quite high because simple, low cost and acceptable tools to monitor temperature continuously are scarce^17,18^. The WHO suggests combining two-site palpation (foot and abdomen) to detect cold stress and hypothermia. However, this method was unreliable, as shown in a study by Ellis and co-workers^19^. In addition, using touch led to an underestimation of neonatal hypothermia^20^. Newer reliable methods for the detection of hypothermia through various hypothermia detection devices have become a priority.

Maintenance of normothermia in preterm and low birth weight neonates is vital for neonatal survival. However, even when all precautions are taken, there were still incidents of drop in temperature below 36.5 °C that would have been undetected for hours or longer without the BEMPU bracelet device, as shown by the current study.

In the current study, despite advice about KMC and education about hypothermia, drop in temperature episodes were detected by the BEMPU device. Thus, this study reveals only the tip of the iceberg of undiagnosed low body temperature in neonates. In a non-research clinical setting, it is likely that there might be more episodes of neonatal hypothermia. Data from the current study thus demonstrate the need for the BEMPU bracelet in detecting temperature drops below 36.5 °C. Early diagnosis of hypothermia would help parents intervene earlier and prevent moderate and severe hypothermia complications.

The BEMPU previously underwent validation study at Jawaharlal Institute of Postgraduate Medical Education & Research (JIPMER), Pondicherry, India. The BEMPU had sensitivity of 98.57, specificity of 99.62, a positive predictive value of 83.45%, and a negative predictive value of 99.62%^21^. The BEMPU's high sensitivity makes it a reliable device to screen for hypothermia in the home and hospital environment. The high specificity of the device satisfies parents that their neonates are normothermic when the device displays the blue light. The BEMPU bracelet may help empower parents to move from a simple caregiver role to a nurse-like or decision-making role in making motivated and confident decisions for the care of their children within the home setting.

Furthermore, in a nonrandomized field trial in Rajasthan (India), the BEMPU group had lower mortality (6%) than the non-BEMPU group (14%). Due to poor follow-up and tracking of KMC hours, this study could not report the influence of BEMPU on the number of KMC hours and weight changes between the groups. However, the authors believe that a higher follow-up rate in the BEMPU group (59.58%) vis-à-vis the Control group (31.34%) indicated that BEMPU would have promoted a positive behavior change in the parents on newborn care^22^. The reduction in mortality in the BEMPU group attests to the assumption.

The study from Rajasthan was from tribal belts, and hence underprivileged population was studied. Our institution is the only tertiary care center for neonates in our region, and most of the population that we serve is underserved with poor socioeconomic status. The loss to follow-up was observed in the current study, both from BEMPU and Control groups. It was mainly attributed to longer distances, financial constraints, and social reasons involved in attending weekly follow-up appointments.

Unlike the current study and the study from Rajasthan, similar RCT from Australia was terminated early as interim analysis showed that there were very few hypothermia events occurring in both groups. This limits the possibility of the BEMPU device being of any additional benefit in resource-rich settings where hypothermia events are rare^23^.

In addition to hypothermia prevention, KMC also increases exclusive breastfeeding, weight, length, and head circumference^24,25^, but in the present study, we did not find any significant differences in weight and length gain. Surprisingly, the mean gain in head circumference was less in the BEMPU group. This may be because both groups received daily average KMC hours of less than 6 h^25^. In a hospital-based study from New Delhi, weight gain was more (6.74 g vs. 3.28 g) in the BEMPU group although it was used for an average of fewer than 4.5 days in the hospital and there were no differences in KMC hours and the number of hypothermia episodes between the two groups^26^.

There are certain limitations of the study. Significant proportion of newborns were dropouts in the study. Home visits by the authors were not done to assess the socio-demographic and environmental factors. Verbal autopsy of causes of death was not done.

The findings of this single-center study suggest that in controlled environments like hospital settings where periodic temperature monitoring is done in addition to clinical examination, the utility of such devices need to be further studied at varied setups. Further research studies are also required to understand the perception and extent of the problem and the feasibility and effectiveness of temperature measurement devices for preventing newborn morbidity and mortality at the community level. The authors strongly suggest replication of the study in different regions of India and across the globe to assess the effectiveness of BEMPU in improving KMC hours along with feasibility and economic analysis before implementing the same.

### Methods

A parallel-group, randomized control trial with equal allocation was conducted from August 2018 to June 2019 (before the onset of COVID-2019 pandemic) at a level 3B NICU of rural tertiary care, a university-affiliated teaching institute in India. The Institute Ethics Committee-2 of HM Patel Centre for Medical Care and Education, Karamsad approved this study via document dated 05/06/2018 and identified by IEC/HMPCMCE/95/Faculty/10/11/18. Informed consent was obtained from parents/legal guardians. The research involving human research participants was carried out in accordance with the Declaration of Helsinki.

### Participant selection

Neonates receiving the care in the step-down nursery were assessed for eligibility two to three days before the time of discharge. Neonates weighing between 1000 and 2000 g irrespective of gestation, who received KMC on three separate days during their stay in the hospital, whose parents received a KMC training program on two separate days of the hospital stay (Video and power-point presentation and counseling about KMC) and willing to return to the hospital for weekly follow-ups for four weeks after discharge were included in the study. Neonates with congenital anomalies, parents not comfortable entering the details in the KMC / Beep chart, using a room heater at home, and not having physical space to provide KMC at home were excluded from the study.

### Outcomes

Primary Outcome: To assess whether constant use of BEMPU improves the duration of KMC at home by at least one hour/day compared to a control group.

Secondary Outcome: To compare the improvement of weight, occurrence of any morbidity, and incidence of hypothermia in the BEMPU group (as recorded by the total number of orange beeps per day).

### BEMPU training

After randomization, the BEMPU was provided to the parents free of cost. It was applied to the wrist of each newborn in the BEMPU group two days before the discharge. This ensured that the parents get accustomed with it. After wearing BEMPU and making it "on" by removing the tag, it shows blue light when the neonate is normothermic. If shows an orange light with a beep alarm sound immediately when skin temperature drops below 36.5 °C. Parents were advised to keep the BEMPU in place 24X7 for 28 days at home and to provide KMC every time when it beeped in addition to the routine KMC at home. They were also advised to consult a doctor immediately if it alarmed continuously, even after starting KMC.

### Study measurements

Parents of neonates in the control group and BEMPU group were taught to enter the details of KMC provided during the last two days of hospitalization in the printed copies of the "KMC chart" (Supplementary material 1). Parents were taught to enter KMC hours in the KMC chart in date and time format only if the individual KMC session was of one hour or more. In addition to the" KMC chart", parents of neonates in the BEMPU group also received "BEMPU beep chart" (Supplementary material 2) during the last two days of hospitalization, in which parents were taught to maintain the beep record of BEMPU. The authors prepared both charts with clear instructions in the vernacular (Gujarati) language. Parents with respect to their group of enrollments received new printed copies "BEMPU beep chart" and/ or "KMC chart" at discharge to be filled on real-time basis at home for the next four weeks.

Potential confounders were taken care of by keeping discharge instructions, KMC education, breastfeeding advice, follow-up schedule, and data collection methods similar between the two groups. Parents were taught about the clothing and wrapping of the newborns. They were also advised to keep the room draught free and to practice bedding in. Weekly follow-ups were held with parents for four weeks after discharge to record measurements (weight, length, head circumference) of growth and daily sessions of KMC at a pediatric outpatient department of the same institute.

The same digital weighing scale [Make: Citizen Weighing System (CWS)], infantometer [seca 416 (Make: Seca, Medical measurement systems and scales), and non-stretchable measure tape were used for the weight (g), length (cm), and head circumference (cm) measurements respectively. The measurements were done in accordance with standard guidelines^27^. The biomedical engineering team did periodic calibration of the weighing scale. The manufacturer calibrated the BEMPU devices.

### Sample size

In the absence of data on KMC at home and the variability in KMC duration, we assumed an effect size of 0.5 for comparing the BEMPU vs. Control group. With an effect size of 0.5, 63 participants were required in each group for 80% power and 5% type I (Alpha) error. Considering that we may not have much loss to follow-up, a sample size of 128 neonates for the study was finalized, with 64 in each group for an effect size of 0.5.

### Randomization

Randomization was done by a statistician who was not a part of the study. Computer-generated random codes were sealed in opaque envelopes. After obtaining written and informed consent from the parents two days before anticipated discharge, newborns were randomized to the BEMPU or control group. Due to the nature of the study, blinding was not possible, but the statistician was blinded about the groups during the analysis.

### Statistical analysis

Data from the charts were transcribed to Office Excel by the study authors (VTP and DVP). Descriptive statistics [mean (SD), frequency (%), median (IQR)] were used to depict the baseline characteristics of the study populations. The Shapiro–Wilk test was used to check the normality of the data. Per protocol analysis was used. Wilcoxon rank-sum test (Mann–Whitney two-sample statistic) was used to assess the median differences between the two groups for non-normal data. Pearson chi-squared test was used two compare categorical variables. An independent sample t-test was used to compare the mean difference of KMC hours and weight gain between groups. Summation of time (hours) of all the individual KMC sessions in 24 h was done to calculate daily KMC hours. Newborns in BEMPU group were also divided in two sub-groups based on whether beeping of the BEMPU ever occurred over four weeks or not to compare the daily KMC hours between these two sub-groups to judge whether beeping promotes KMC or not. Similarly, daily KMC hours were also compared between one of the sub-groups of BEMPU group in which there were no beeps ever versus daily KMC hours in control group. Analysis was performed using STATA 14.2 and a p-value less than 0.05 was considered statistically significant.

### Trial registration

Clinical Trials Registry India, registration number: CTRI/2018/08/015,154 dated 01/08/2018 and accessible at http://ctri.nic.in/Clinicaltrials/pdf_generate.php?trialid=27600&EncHid=&modid=&compid=%27,%2727600det%27.

## Supplementary Information

Supplementary Information 1.

Supplementary Information 2.

## Data Availability

The data can be obtained from the corresponding author on request by email.
