# Supplementary material for: Impact of hypothermia alert device (BEMPU) on improvement of duration of Kangaroo Mother Care (KMC) provided at home: parallel-group randomized control trial
Source: Sci Rep. 2023 Mar 16;13:4368. doi: 10.1038/s41598-023-29388-0 (PMC10020158; doi:10.1038/s41598-023-29388-0)
Supplement: Supplementary file 1 — Supplementary Information 1. [file 41598_2023_29388_MOESM1_ESM.pdf]

Mother / Legally authorized representative (LAR) to fill at home

Participant Number:

1. Who provides KMC at home?

2. Tick in the KMC chart when you provide KMC on daily basis

3. Come to Hospital on these dates and days

    1.

    2.

    3.

    4.

4. Call on this number if any help needed

Name:

Mobile:

Mother / Legally authorized representative (LAR) to fill at home

Participant Number:

Kangaroo Mother Care record: Tick at appropriate level

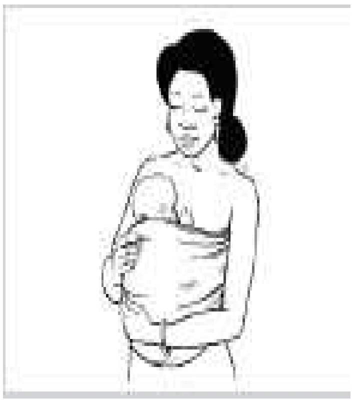

WEEK 1: Day hours

DATE/ TIME	08:00: AM	9	10	11	12	01:00 PM	2	3	4	5	6	7

WEEK 1 : Night hours

DATE/ TIME	08:00: PM	9	10	11	12	01:00 AM	2	3	4	5	6	7

Mother / Legally authorized representative (LAR) to fill at home

Participant Number:

Kangaroo Mother Care record: Tick at appropriate level

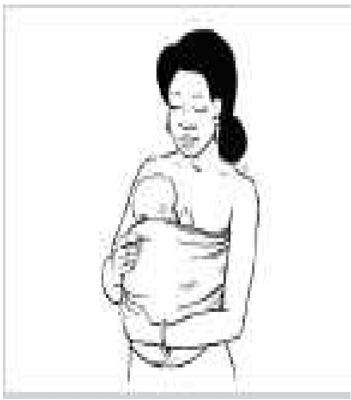

WEEK 2: Day hours

DATE/ TIME	08:00 AM	9	10	11	12	01:00 PM	2	3	4	5	6	7

WEEK 2: Night hours

DATE/T IME	08:0 0PM	9	10	11	12	01:00 AM	2	3	4	5	6	7

Mother / Legally authorized representative (LAR) to fill at home

Participant Number:

Kangaroo Mother Care record: Tick at appropriate level

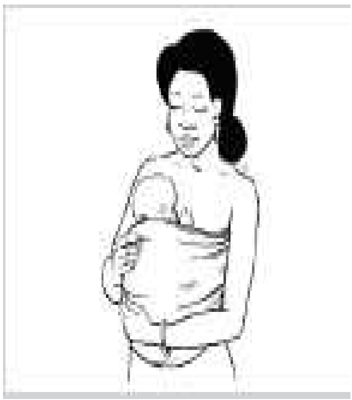

WEEK 3 : Day hours

DATE/ TIME	08:00 AM	9	10	11	12	01:00 PM	2	3	4	5	6	7

WEEK 3: Night hours

DATE/ TIME	08:00 PM	9	10	11	12	01:00 AM	2	3	4	5	6	7

Mother / Legally authorized representative (LAR) to fill at home

Participant Number:

Kangaroo Mother Care record: Tick at appropriate level

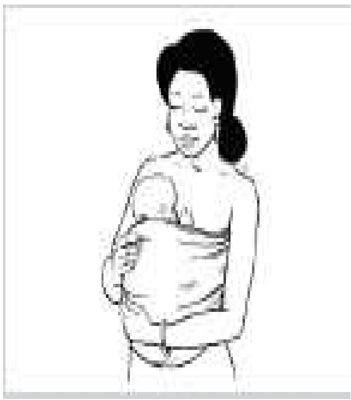

WEEK 4: Day hours

DATE/ TIME	08:00 AM	9	10	11	12	01:00 PM	2	3	4	5	6	7

WEEK 4 : Night hours

DATE/ TIME	08:00 PM	9	10	11	12	01:00 AM	2	3	4	5	6	7
