# Supplementary material for: Impact of hypothermia alert device (BEMPU) on improvement of duration of Kangaroo Mother Care (KMC) provided at home: parallel-group randomized control trial
Source: Sci Rep. 2023 Mar 16;13:4368. doi: 10.1038/s41598-023-29388-0 (PMC10020158; doi:10.1038/s41598-023-29388-0)
Supplement: Supplementary file 2 — Supplementary Information 2. [file 41598_2023_29388_MOESM2_ESM.pdf]

Mother / Legally authorized representative (LAR) to fill at home

BEMPU orange beep record: Tick at appropriate level

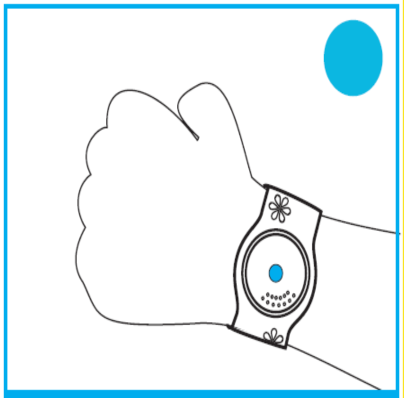
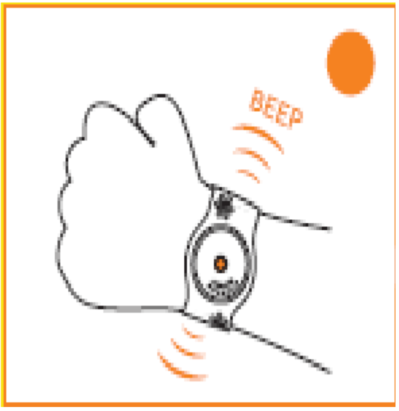

WEEK 1: Day hours

DATE/ TIME	08:00 AM	9	10	11	12	01:00 PM	2	3	4	5	6	7

WEEK 1 : Night hours

DATE/ TIME	08:00 PM	9	10	11	12	01:00 AM	2	3	4	5	6	7

Mother / Legally authorized representative (LAR) to fill at home

BEMPU orange beep record: Tick at appropriate level

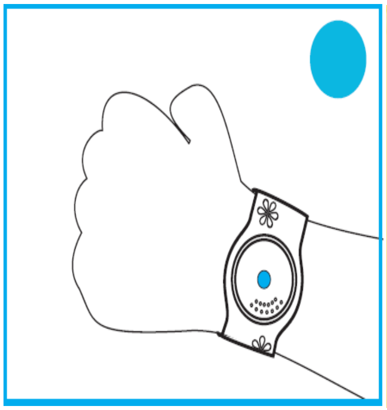

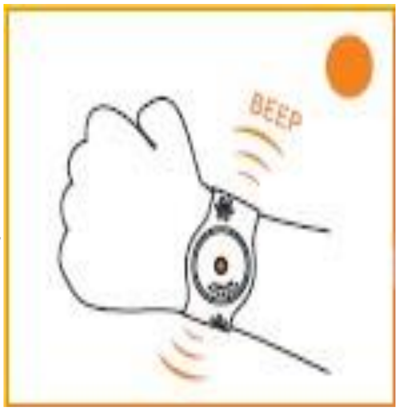

WEEK 2: Day hours

DATE/ TIME	08:00 AM	9	10	11	12	01:00 PM	2	3	4	5	6	7

WEEK 2: Night hours

DATE/ TIME	08:00 PM	9	10	11	12	01:00 AM	2	3	4	5	6	7

Mother / Legally authorized representative (LAR) to fill at home

BEMPU orange beep record: Tick at appropriate level

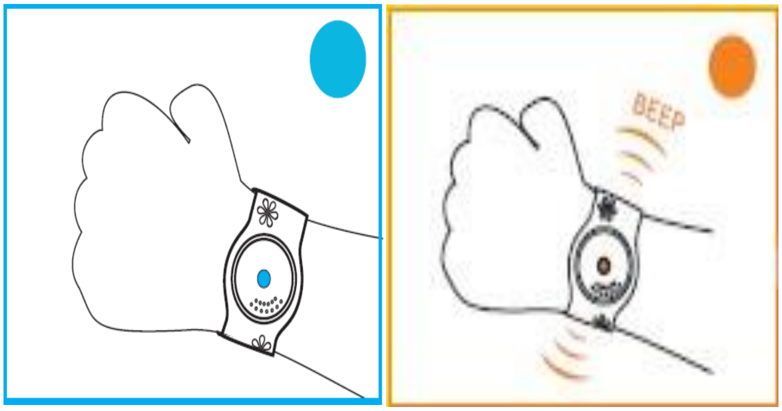

WEEK 3 : Day hours

DATE/ TIME	08:00 AM	9	10	11	12	01:00 PM	2	3	4	5	6	7

WEEK 3: Night hours

DATE/ TIME	08:00 PM	9	10	11	12	01:00 AM	2	3	4	5	6	7

Mother / Legally authorized representative (LAR) to fill at home

BEMPU orange beep record: Tick at appropriate level

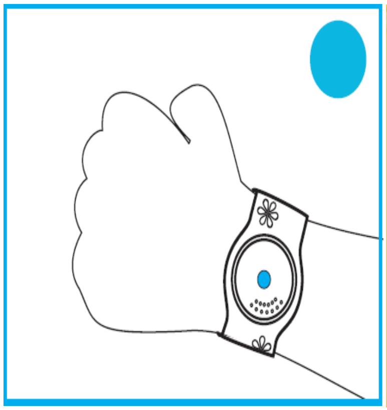

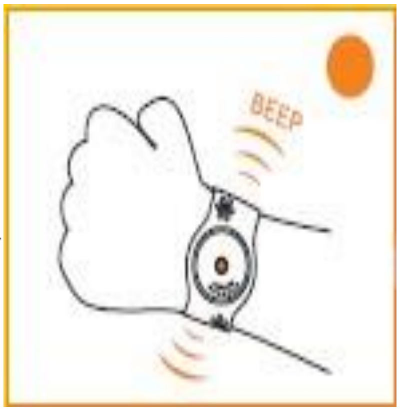

WEEK 4 : Day hours

DATE /TIME	08:00: AM	9	10	11	12	01:00 PM	2	3	4	5	6	7

WEEK 4 : Night hours

DATE/ TIME	08:00 PM	9	10	11	12	01:00 AM	2	3	4	5	6	7

Mother / Legally authorized representative (LAR) to fill at home

BEMPU orange beep record: Tick at appropriate level

Operating instructions for BEMPU and Kangaroo Mother Care

1. Normal body temperature is important for health and optimum growth of a newborn.

2. To maintain the normal body temperature of a Low Birth Weight newborn, give Kangaroo Mother Care.

3. Apply the BEMPU on the wrist of the newborn, so that it remains in contact with the skin.

3. Many times newborn's temperature drops, which parent can not recognize. This BEMPU device gives orange light and beeping sound immediately when newborn's temperature drops.

4. Whenever there is an orange light and beeping sound from BEMPU, Kangaroo Mother Care becomes essential. While giving Kangaroo Mother Care, apply cap, socks and gloves to the newborn. After few minutes of starting Kangaroo Mother Care, orange light will change to blue light and beeping sound will stop. This indicates that the newborn's temperature has become normal. At this moment, Kangaroo Mother Care should not be stopped, it should be continued.

5. You will be able to use BEMPU for one month continuously.

6. After one month, there will be white light from the BEMPU and then it will stop functioning.

7. Return the BEMPU when you come for follow up at the end of fourth week.
